# A novel technique for gastrostomy tube replacement following post-radiotherapy esophageal atresia: the joint forces of double endoscopy

**DOI:** 10.1055/a-2532-4514

**Published:** 2025-03-21

**Authors:** Zeliang Yang, Yong Liu, Shun He, Hongqing Li, Wang Guiqi

**Affiliations:** 1 12501 National Cancer Center/National Clinical Research Center for Cancer /Cancer Hospital, Department of Endoscopy, Chinese Academy of Medical Sciences & Peking Union Medical College, Beijing, China; 2 680061 Department of Gastroenterology, Anqing Municipal Hospital, Anqing, China

Percutaneous endoscopic gastrostomy provides a safe and effective minimally invasive surgical route, thus ensuring long-term enteral nutritional support therapy 1 . Patients with esophageal cancer may develop scars from long-term radiotherapy, causing complete esophageal obstruction and difficulties in replacing gastrostomy tubes. Our center utilizes a new approach to replacing gastrostomy tubes by incising the atretic segment of the esophagus through transoral and transgastric fistula bimodality ( Video 1 ).

The joint forces of double endoscopy in the replacement of a gastrostomy tube.Video 1

A 64-year-old woman was diagnosed with squamous cell carcinoma at the entrance of the esophagus 12 years previously, and then underwent continuous radiation therapy a total of 33 times, after which esophageal stenosis with dyspnea and paralysis of the vocal cords occurred. A tracheotomy was performed. Two years ago, endoscopic excision of the esophageal stenosis scar was performed and percutaneous endoscopic gastrostomy was performed. Repeat endoscopic excision of the esophageal stenosis scar and endoscopic dilation using a Savary dilator were performed because of recurrence of the esophageal stenosis. Then, 1 month ago, endoscopy suggested esophageal atresia and the need to replace the gastrostomy tube ( Fig. 1 ).

**Fig. 1:**
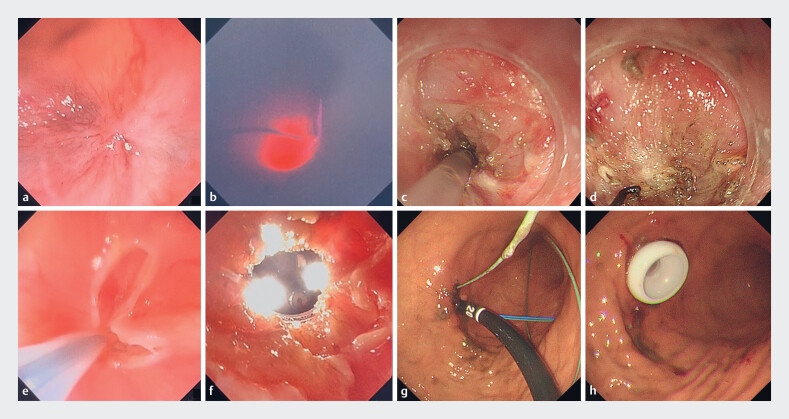
Gastrostomy tube replacement procedure. **a** Esophageal atresia was seen on endoscopy. **b** Transillumination test was employed to assist in clarifying the direction of dissection. **c, d** Oral-side endoscopy: a knife was used to incise the scar and a syringe needle was used to further define the direction of the incision. **e, f** Anal-side endoscopy: the scar was incised and the two endoscopes successfully rendezvoused. **g, h** Successful replacement of gastrostomy tube.

First, esophageal atresia was viewed endoscopically, but the guidewire could not be passed ( Fig. 1 **a** ). An ultra-fine endoscope was applied along the sinus tract of the gastrostomy to enter the stomach and then into the esophagus, and complete atresia of the esophageal lumen was observed, with a positive transillumination test to assist in clarifying the direction of dissection ( Fig. 1 **b** ). Oral-side endoscopy was performed, using a knife to incise the scar and a syringe needle for injection of methylene blue to further define the direction of the incision ( Fig. 1 **c, d** ). Anal-side endoscopy was performed, using a snare to incise the scar, and the two endoscopes successfully rendezvoused ( Fig. 1 **e, f** ). Finally, the guidewire was sought for gastrostomy tube implantation ( Fig. 1 **g, h** ).

The double endoscopy-assisted gastrostomy tube replacement technique is a safe and effective method of replacing fistulas for esophageal atresia. Further research and clinical experience are required.

Endoscopy_UCTN_Code_TTT_1AO_2AK

## Notes

Endoscopy E-Videos https://eref.thieme.de/e-videos
*E-Videos* is an open access online section of the journal *Endoscopy* , reporting on interesting cases and new techniques in gastroenterological endoscopy. All papers include a high-quality video and are published with a Creative Commons CC-BY license. Endoscopy E-Videos qualify for HINARI discounts and waivers and eligibility is automatically checked during the submission process. We grant 100% waivers to articles whose corresponding authors are based in Group A countries and 50% waivers to those who are based in Group B countries as classified by Research4Life (see: https://www.research4life.org/access/eligibility/ ). This section has its own submission website at https://mc.manuscriptcentral.com/e-videos .

## References

[1] Farrag K Shastri YM Beilenhoff U Percutaneous endoscopic gastrostomy (PEG): a practical approach for long term management BMJ 2019 364 k5311 10.1136/bmj.k5311 30670385

